# The Rattlesnake Poison

**Published:** 1866-02

**Authors:** 


### THE RATTLESNAKE POISON.

To ascertain the power and amount of this poison, Dr. Dearing performed the following experiment:

The snake was a very large and vicious one, and very active at the time. He took eight half-grown chickens, and allowed the snake to strike at each, under the wing, as fast as they could be presented to him. The first died immediately; the second, after a few minutes; the third, after ten minutes; the fourth, after more than an hour; the fifth, after twelve hours; the sixth was sick and drooping for several days, but recovered; the seventh was only slightly affected, and the eighth not at all.

With my second remaining specimen, I was desirous of performing several experiments as to the action of this poison on the blood. The following is one:—The snake was quite active, and as any one approached the cage, began to rattle violently; but twenty-five or thirty drops of chloroform being allowed to fall on his head, one slowly after the other, the sound of his rattle gradually died away, and in a few minutes he was wholly under the effects of this agent. He was then adroitly seized behind the jaws with the thumb and forefinger, and dragged from the cage and allowed to partially resuscitate; in this state a second person held his tail to prevent him coiling around the arm of the first, while a third opened his mouth and with a pair of forceps pressed the fang upward, causing a flow of the poison, which was received on the end of a scalpel. The snake was then returned into the cage. Blood was then extracted from a finger for microscopical examination. The smallest quantity of the poison being presented to the blood between the glasses, a change was immediately perceived—the corpuscles ceased to run and pile together, and remained stagnant, without any special alteration of structure; the whole appearance was as though the vitality of the blood had been suddenly destroyed, exactly as in death from lightning. This agrees also with another experiment, performed on a fowl, where the whole mass of the blood appeared quite liquid, and having little coagulable power. The physiological action of this poison on animals is probably that of a most powerful sedative, acting through the blood on the nervous centres. This is shown by the remarkable fact that its full and complete antidotes are the most active stimulants. Of these, alcohol in some shape is the first. (Dr. W. J. BURNETT, before the Boston Natural History Society—*Things not Generally known* and *Dental Cosmos.*)

